# Corrosion Resistance of AISI 316L Stainless Steel Biomaterial after Plasma Immersion Ion Implantation of Nitrogen

**DOI:** 10.3390/ma14226790

**Published:** 2021-11-10

**Authors:** Viera Zatkalíková, Juraj Halanda, Dušan Vaňa, Milan Uhríčik, Lenka Markovičová, Milan Štrbák, Lenka Kuchariková

**Affiliations:** 1 Department of Materials Engineering, Faculty of Mechanical Engineering, University of Žilina, Univerzitná 8215/1, 010 26 Žilina, Slovakia; milan.uhricik@fstroj.uniza.sk (M.U.); lenka.markovicova@fstroj.uniza.sk (L.M.); milan.strbak@fstroj.uniza.sk (M.Š.); lenka.kucharikova@fstroj.uniza.sk (L.K.); 2 Faculty of Materials Science and Technology, Advanced Technologies Research Institute, Slovak University of Technology in Bratislava, Jána Bottu Č. 2781/25, 917 24 Trnava, Slovakia; juraj.halanda@stuba.sk (J.H.); dusan.vana@stuba.sk (D.V.)

**Keywords:** plasma immersion ion implantation of nitrogen, austenitic stainless steel, corrosion resistance, potentiodynamic polarization, electrochemical impedance spectroscopy

## Abstract

Plasma immersion ion implantation (PIII) of nitrogen is low-temperature surface technology which enables the improvement of tribological properties without a deterioration of the corrosion behavior of austenitic stainless steels. In this paper the corrosion properties of PIII-treated AISI 316L stainless steel surfaces are evaluated by electrochemical impedance spectroscopy (EIS), potentiodynamic polarization (PP) and exposure immersion tests (all carried out in the 0.9 wt. % NaCl solution at 37 ± 0.5 °C) and compared with a non-treated surface. Results of the three performed independent corrosion tests consistently confirmed a significant increase in the corrosion resistance after two doses of PIII nitriding.

## 1. Introduction

Austenitic stainless steels are widely used biomaterials owing to their high biocompatibility and corrosion resistance. Their current study mostly aims to further improve their mechanical properties, wear and local corrosion resistance [1,2,3,4].

Plasma nitriding process is one of the methods for the thermochemical treatment of austenitic stainless steels to improve the tribological properties of their surfaces. Conventional plasma nitriding performed at the temperatures of above 500 °C ensures a high resistance to wear, however, the precipitation of chromium nitride causes a depletion of chromium in the solid solution and this leads to a reduction in the corrosion resistance. Unlike this technology the plasma immersion ion implantation of nitrogen (PIII) is performed at temperatures below 400 °C and it enables the thermochemical surface treatment with a marked increase of hardness and wear resistance without a deterioration of the corrosion behaviour of stainless steel [1,2,5,6,7]. According to numerous authors [1,5,6,7,8,9,10,11,12,13,14], so-called expanded austenite is the phase formed in the N-modified layer under low-temperature conditions and responsible for the improved properties of such treated stainless steels. According to the authors of [10], expanded austenite is a crystalline cubic phase with considerably expanded austenitic lattice which may contain precipitates of different size and quantity depending on the nitriding temperature used.

PIII technology is based on immersion of the specimen in a plasma and applying negative high-voltage pulses to it. Positively charged ions are extracted from the plasma through the plasma sheath and they are implanted on the whole surface at the same time for reduction of the process time for large pieces and for decreasing the costs [2]. PIII allows one to obtain a higher current density of ions with significantly shorter implantation times and it is also applicable for workpieces with complicated surface shapes [15].

Some authors have studied the corrosion resistance of PIII nitrided stainless steel surfaces and found improved pitting corrosion resistance in variously concentrated chloride environments [1,8,11,12,13]. In contrast, other authors [7] reported different results for AISI 304 stainless steel in 1 wt. % NaCl solution (PIII treated at 300–380 °C).

Regarding biomedical applications, the authors of [11] recommend low-temperature nitriding for improvement of tribological and corrosion properties of 316L stainless steel implants and for the prevention of metal release as well. The corrosion resistance increase was recorded in aerated phosphate-buffered saline solution by PP measurements [11]. Similarly, the authors of [16] confirmed an increase of the pitting corrosion resistance of nitride-coated AISI 316L coronary stents, indicated by PP method and long-term immersion (6 months) in simulated body fluid at 37 ± 1 °C.

According to one study [1], the higher resistance to pitting after PIII nitriding of AISI 316LVM stainless steel (by PP in 3.5 wt. % NaCl solution) can be explained by an increase of the pitting resistance equivalent number (PREN = wt. % Cr + 3.3 × wt. % Mo + 16 × wt. % N) in the surface film. The authors also confirmed a close relation between PREN and the pitting potential.

The objective of the presented paper is to compare the corrosion resistance of PIII nitrided AISI 316L stainless steel surfaces with that of an original non-treated (as received) surface. The evaluation is based on three independent test methods: electrochemical impedance spectroscopy (EIS), potentiodynamic polarization and exposure immersion test, all carried out in a 0.9 wt. % NaCl solution at the temperature of 37 ± 0.5 °C for simulation of the internal environment of a human body. The nitrided surfaces were also characterized by SEM and EDX analysis.

## 2. Materials and Methods

**Figure 1:**
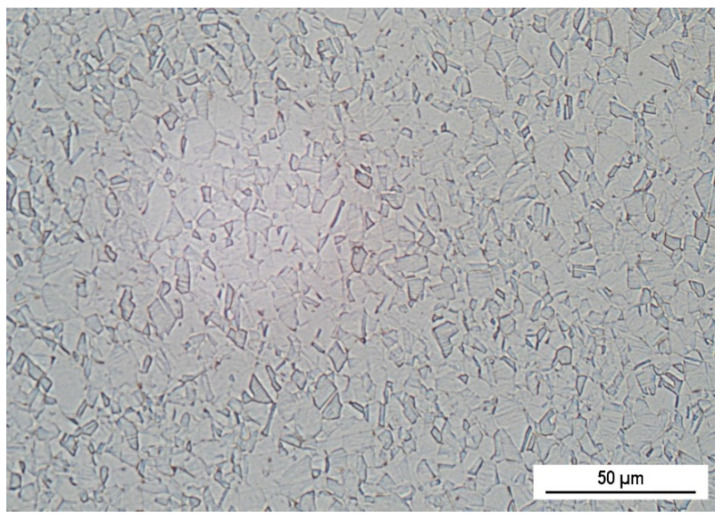
Microstructure of AISI 316L stainless steel, longitudinal section (Kallings 2 etch.).

The experimental material AISI 316L is Cr-Ni-Mo austenitic stainless steel (wt. %: Cr 16.97, Ni 14.75, Mo 2.50, Mn 1.76, C 0.03, Si 0.49, Cu 0.15, Fe balance) purchased in the form of rectangular specimens 15 mm × 40 mm × 2 mm (BEZNOSKA Slovakia Ltd., Banská Bystrica, Skovakia). Its microstructure (Figure 1) is formed by polyhedral austenitic grains with numerous twins, which could be created by annealing or by rolling.

PIII nitriding of experimental specimens was performed in the specialized Laboratory of Plasma Technologies in the Advanced Technologies Research Institute (Slovak University of Technology Bratislava, Faculty of Materials Science and Technology in Trnava, Slovakia).

Prior to nitriding, the surface of the specimens was not mechanically or chemical-ly treated and only degreased with ethanol. The specimens were placed on a holder con-nected to a high voltage power supply and electrically isolated from the chamber wall. The nitriding took place in two stages.During the first stage, the same dose of nitrogen (5 × 10^17^ at/cm^2^) was used in all samples. In the second stage a half of specimens was nitrided by one more dose of the nitrogen (5 × 10^17^ at/cm^2^) at the same nitriding conditions. The result was two types of nitrided surfaces: with one applied dose of nitrogen (1N) and with two applied doses (2N). An overview of the tested surfaces and specimen designations for the experiments is given in Table 1.

**Figure 2:**
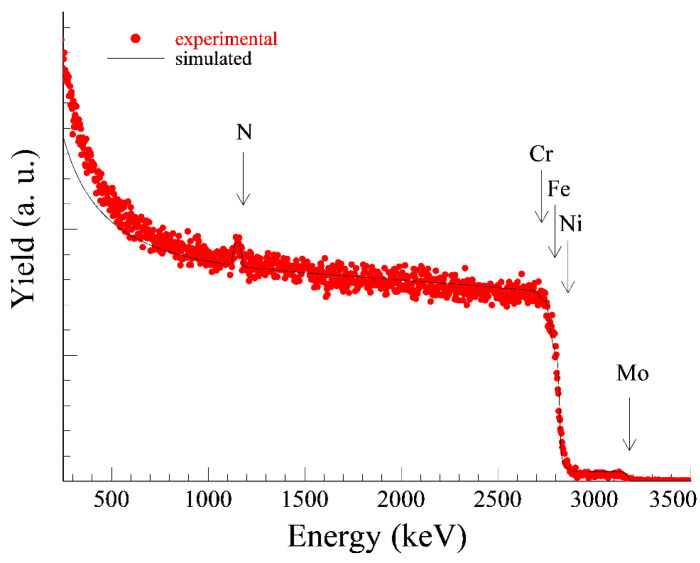
Measured and evaluated RBS spectrum of 1N specimen, measured N dose 2.7 × 10^17^ at/cm^2^.

**Figure 3:**
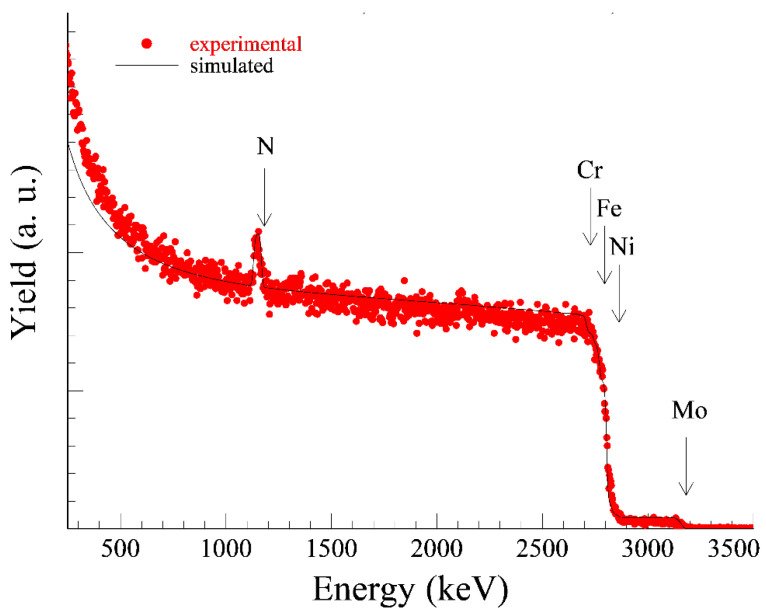
Measured and evaluated RBS spectrum of 2N specimen, measured N dose 4.2 × 10^17^ at/cm^2^.

The size of the implanted doses was chosen according to the recommendations of the authors [1] and [5]. Nitrogen plasma was generated by a radio frequency discharge with a power of 500 W at a chamber pressure of 0.3 Pa. To improve the processing homogeneity the samples were rotated (10 rpm) during the process. The one dose application of nitrogen lasted approximately 5 h. The pulsed accelerating voltage used was 20 kV (due to the technical dispositions of the device) with a frequency of 150 Hz and a pulse length of 15 µs. The Rutherford Back Scattering (RBS) method was used to determine the exact implanted nitrogen dose (Figure 2 and Figure 3).

According to the height of the nitrogen peaks on RBS curves (evaluated by the SIMNRA software) the lower implanted dose (1N) was in fact 2.7 × 10^17^ at/cm^2^ (the setting on the device was 5 × 10^17^ at/cm^2^). The higher implanted dose (2N) was set to 1 × 10^18^ at/cm^2^ (which means 2 × 5 × 10^17^ at/cm^2^) and in fact 4.2 × 10^17^ at/cm^2^ was measured. The differences between the set and actual dose were caused by the so-called dust removal effect.

For more detailed characterization, the nitrided specimen surfaces were displayed and EDX analysed by a Vega scanning electron microscope (SEM, Tescan, Brno, Czech Republic).

The temperature of 37 ± 0.5 °C and 0.9 wt. % sodium chloride solution for simulation the internal environment of the human body was used as the environment for corrosion tests. The electrochemical corrosion tests (EIS and potentiodynamic polarization) were performed in the conventional three-electrode cell system with a calomel reference electrode (SCE) and a platinum auxiliary electrode (Pt) using a BioLogic corrosion measuring system a with PGZ 100 measuring unit (BioLogic, Seyssinet-Pariset, France). The time for potential stabilization between the specimen and the electrolyte was set to 10 min. The exposed area of a specimen was 1 cm^2^.

Electrochemical impedance spectroscopy measurements were recorded at the corrosion potential over a frequency range from 100 KHz to 5 mHz. Results of EIS measurements were displayed as Nyquist curves plotted in coordinates of real and imaginary impedance components. The polarization resistance (R_p_) values were obtained by the analysis of the representative Nyquist curves using the EC-LAB software (BioLogic, Seyssinet-Pariset, France).

The potentiodynamic polarization curves were recorded at the sweep rate of 1 mV/s, a potential scan range was applied between −0.3 and 1.2 V vs. open circuit potential (OCP) [17,18]. For both EIS and PP measurements at least three experiment repeats were carried out for each type of surface.

For 50-days exposure immersion tests specimens of rectangular shape (15 mm × 40 mm × 1.5 mm) were degreased by ethanol and weighted out with accuracy (±0.00001 g). A group of three parallel specimens was tested for each type of surface. After exposure the specimens were carefully brushed, washed with demineralized water, freely dried and weighed again [18].

## 3. Results and Discussion

**Figure 4:**
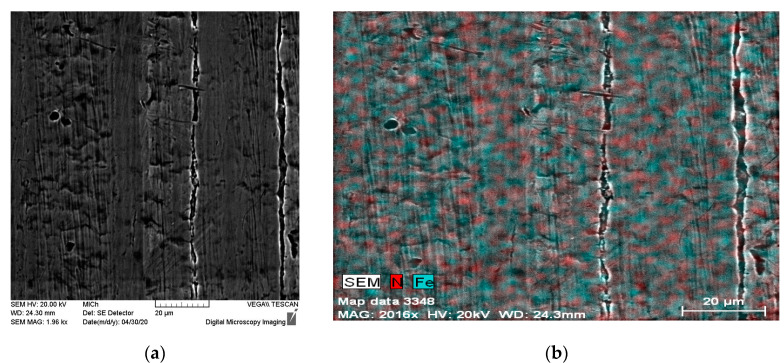
1N nitrided specimen surface (**a**) SEM, (**b**) EDX map—N and Fe distribution.

**Figure 5:**
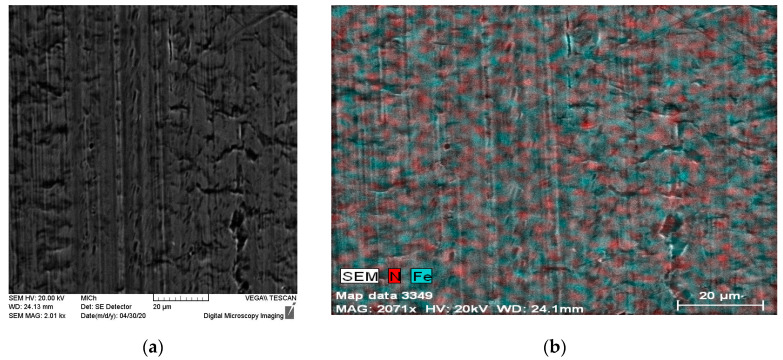
2N nitrided specimen surface (**a**) SEM, (**b**) EDX map—N and Fe distribution.

The nitrided surfaces (1N and 2N) observed by SEM and, also EDX maps expressing N and Fe distribution are shown in Figure 4 and Figure 5. EDX surface analysis revealed an uneven distribution of nitrogen on the surface layer of the tested stainless steel in the case of both 1N and 2N specimens. The same as described in studies with similar conditions of PIII nitriding, nitrogen may be present in the form of expanded austenite [1,2,5,6,8] which is difficult to identify and it is observable using a transmission electron microscope [1,5].

### 3.1. EIS Test

**Figure 6:**
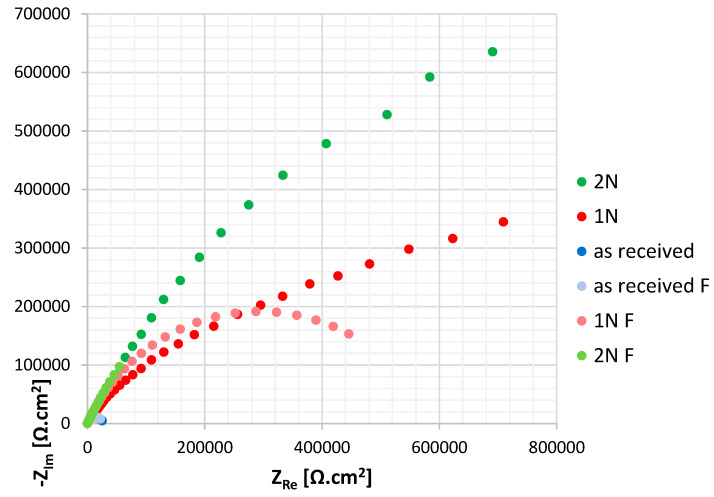
Nyquist curves for tested surfaces (fitted curves are “F” marked).

The measured impedance spectra were simple and therefore a single loop circuit consisting of electrolyte resistance (R_Ω_), polarization resistance (R_p_) and CPE element connected to the circuit instead of the capacitance (R_Ω_ + CPE/R_p_), was used for the Nyquist curves evaluation [19,20,21]. The CPE element was used to simulate inhomogeneities of the surface layer [21]. The Nyquist curves for the tested surfaces are shown in Figure 6, values of the EIS parameters calculated by the EC-LAB software are listed in Table 2.

The polarization resistance (R_p_) enables one to assess the passive film quality: a higher R_p_ value points to a higher quality. As can be seen, the PIII nitriding brought a sharp increase of R_p_ values of both 1N and 2N surfaces. Two-dose nitriding has been shown to be particularly effective: R_p_ value for 2N specimen was more than twice higher than for 1N. High quality passive film of PIII nitrided specimens may be connected to the molybdenium present in the tested AISI 316L stainless steel (2.5 wt. %). According to several authors [12,22,23] Mo atoms tend to stabilize the expanded austenite structure by attracting nitrogenium atoms around themselves and this leads to the prevention of chromium nitride precipitation. Two-dose nitriding could enhance these processes, resulting in a high resistance of a thus-treated surface.

### 3.2. Potentiodynamic Polarization Test

**Figure 7:**
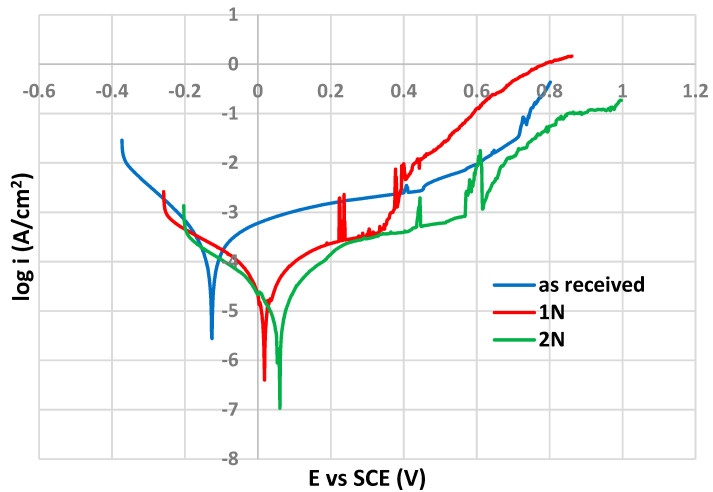
Potentiodynamic polarization curves for tested surfaces.

As shown in Figure 7, the shape of polarization curves is typical for passivating metals (passive anodic branches reflecting the control of the anodic dissolution rate by the passive current density) [24] and therefore the corrosion potential (E_corr_) values were determined directly from the PP curves (Tafel extrapolation was not applicable).

The pitting potential (E_p_) values, that denote the disruption of the passive surface film and the onset of the stable pit growth, were determined as the potentials of a sudden permanent increase in current density after reaching the passivity state. The values of both above mentioned PP parameters are listed in Table 3.

According to the E_corr_ values, PIII nitriding caused a marked increase in the thermodynamic stability of the tested surfaces, and the highest E_corr_ value was reached for 2N surface (0.059 V vs. SCE). An E_corr_ increase of about 0.1 V between non-treated and PIII nitride surfaces (AISI 316L in 5 wt. % NaCl solution) was also recorded by other authors [12]. Olzon-Dionisio et al. [8] observed an E_corr_ difference of 0.15 V for the same surface (AISI 316L in 5 wt. % NaCl solution) after using the same nitriding temperature.

Regarding the evaluation of the pitting corrosion resistance expressed by the E_p_ value (0.330 V vs. SCE), the single dose nitriding (1N) probably did not provide a sufficiently uniform passive film. E_p_ value after 2N nitriding is significantly higher (0.567 vs. SCE). A similar dependence of E_p_ on the PIII nitriding time was observed in the study [11], but not under the same PP experiment conditions (different solution and temperature). The best pitting corrosion resistance of the 2N surface could be related to the role of nitrogen in the corrosion pits repassivation process. According to the authors of [13] nitrogen is responsible for pH increase which facilitates the pits’ repassivation, because of its reduction (from N^0^ to N^−III^) by binding of protons H^+^ into ammonium cations (NH_4_^+^). Subsequently, NH_4_^+^ cations can undergo oxidation to NO_2_^−^ anions [13] which can contribute to pit repassivation [13]. These processes could be affected by the content of nitrogen in the steel surface film.

### 3.3. Exposure Immersion Test

**Figure 8:**
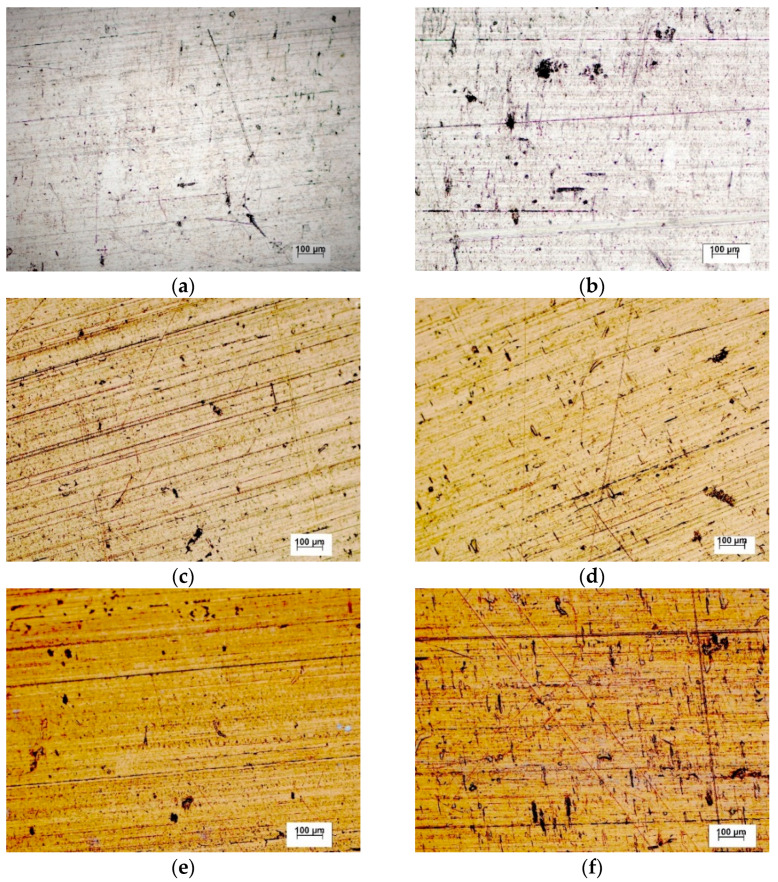
The tested stainless steel surfaces before and after 50-days exposure (optical microscope): as received surface (**a**) before, (**b**) after; 1N surface (**c**) before, (**d**) after; 2N surface (**e**) before, (**f**) after.

The tested stainless steel surfaces before and after 50-days exposure under conditions simulating the internal human body environment (0.9 wt. % NaCl solution at 37 °C) are shown in Figure 8. The average corrosion rates calculated from the mass losses of the specimens (mass loss per unit area per unit time, g/(m^2^ day)) are listed in Table 4.

After performing the exposure tests, all three types of surfaces (as received, 1N, 2N) showed only very small differences in appearance compared to the state before. According to the values of average corrosion rates, the pitting seemed to be most pronounced on the surface of the as received specimens, where is probably related to the sites of mechanical damage with an imperfect passive film. Pitting corrosion of austenitic stainless steels initiated in this way has been documented by several authors, e.g., [18,21,25]. The average corrosion rates values of 1N and 2N specimens are less than half compared to the as received one. This corresponds with the results of both electrochemical corrosion tests and points to the abovementioned positive role of nitrogen in the pit repassivation process (Section 3.2). Recorded low corrosion rates also indicate a potentially negligible leakage of harmful substances into the human body [26,27,28,29,30] and suggest a high biocompatibility of PIII nitrided surfaces [11].

## 4. Conclusions

On the bases of the performed experiments the following can be concluded:

- Surface EDX analysis of 1N and 2N specimens revealed uneven distribution of nitrogen.
- According to the R_p_ values of 1N and 2N surfaces, PIII nitriding brought about a significant increase of the passive film quality. Two-dose nitriding resulted in a more than two-fold higher R_p_ than recorded for 1N.
- The results of the PP test showed that the surface after two-dose PIII nitriding (2N) appeared to be the most resistant to pitting (this was shown by the highest E_corr_ and E_p_ potential values). This is probably associated with a positive role of nitrogen in the repassivation process.
- Average corrosion rates calculated from mass losses during exposure immersion test confirmed the high corrosion resistance of both 1N and 2N surfaces.

According to the obtained experiment results, PIII nitriding appears to be a suitable method for the surface treatment of austenitic stainless steels implants. However, before actual biomedical applications deeper studies of the relations between the surface roughness before PIII nitriding and the final biocompatibility (including the corrosion resistance and resistance to the biofilm formation) would be beneficial.

## Figures and Tables

**Table 1:** Overview of tested surfaces.

Type of Surface	Specimen Designation
PIII nitrided by one dose (5 × 10^17^ at/cm^2^)	1N
PIII nitrided by two doses (2 × 5 × 10^17^ at/cm^2^)	2N
Original non-treated	as received

**Table 2:** Values of EIS parameters.

Specimen Designation (Type of Surface)	Polarization Resistance R_p_ (kΩ.cm^2^)	Electrolyte Resistance R_Ω_ (kΩ.cm^2^)
As received	27.5 ± 0.3	0.054 ± 0.002
1N	587.1 ± 1.5	0.040 ± 0.001
2N	1256.0 ± 1.9	0.057 ± 0.002

**Table 3:** Values of PP parameters.

Specimen Designation (Type of Surface)	Corrosion Potential E_corr_ (V vs. SCE)	Pitting Potential E_p_ (V vs. SCE)
as received	−0.126 ± 0.02	0.449 ± 0.03
1N	0.017 ± 0.03	0.330 ± 0.04
2N	0.059 ± 0.02	0.567 ± 0.03

**Table 4:** Average corrosion rates calculated from mass losses during the exposure test.

Specimen Designation (Type of Surface)	Average Corrosion Rate (g/(m^2^ day))
As received	0.00094 ± 0.93%
1N	0.00038 ± 0.81%
2N	0.00033 ± 0.84%

## Data Availability

Data sharing is not applicable to this article.
